# Potassium Replacement Practices and Their Association With Blood Transfusion Outcomes in Surgical and Critical Care Patients: A Systematic Review

**DOI:** 10.7759/cureus.84978

**Published:** 2025-05-28

**Authors:** Muhammad Yousuf, Jarallah H. J. Alkhazendar, Aliaa H Alkhazendar, Syed Muhammad Baqar Raza, Maria Javed, Soban Haider, Shahzad Ahmad

**Affiliations:** 1 Acute Medicine, Kingston Hospital, Kingston and Richmond NHS Foundation Trust, London, GBR; 2 General and Emergency Surgery, Lister Hospital, East and North Hertfordshire NHS Trust, Stevenage, GBR; 3 Surgery, Islamic University of Gaza, Gaza City, PSE; 4 Cardiac Surgery, Rawalpindi Institute of Cardiology, Rawalpindi, PAK; 5 Vascular Surgery, Evangelisches Krankenhaus Hubertus, Berlin, DEU; 6 Medicine, Medcare International Hospital, Gujranwala, PAK; 7 Medicine, National University Of Medical Sciences, Rawalpindi, PAK; 8 Medicine, Islamic International Medical College, Riphah International University, Rawalpindi, PAK; 9 Surgery, Liaquat National Hospital, Karachi, PAK

**Keywords:** blood transfusion, critical care, electrolyte imbalance, hemoglobin, hypokalemia, icu, perioperative management, potassium replacement, surgery, transfusion outcomes

## Abstract

This systematic review examines the association between potassium replacement strategies and blood transfusion outcomes in surgical and critical care settings. Despite the frequent use of potassium supplementation to address hypokalemia in hospitalized patients, its direct and indirect impact on transfusion requirements remains underrecognized. A structured literature search identified five eligible studies that investigated both potassium management and transfusion outcomes across diverse high-acuity populations. Findings revealed that aggressive or routine electrolyte monitoring and replacement may contribute to iatrogenic anemia through repeated phlebotomy, while preoperative hypokalemia was associated with increased need for transfusion in select cohorts. Although the degree of association varied, several studies suggested that individualized potassium replacement protocols could play a role in optimizing transfusion practices. These insights highlight the importance of integrated electrolyte and transfusion management to improve patient safety and conserve resources in perioperative and intensive care environments.

## Introduction and background

Electrolyte management remains a cornerstone of perioperative and critical care, with potassium being one of the most closely monitored and frequently corrected electrolytes in hospitalized patients [1]. Hypokalemia, arising from surgical stress, fluid shifts, pharmacological agents, or underlying medical conditions, is frequently encountered in both surgical wards and intensive care units (ICUs). Inadequate or overly aggressive potassium replacement has been associated with a spectrum of adverse outcomes, including cardiac arrhythmias, extended hospitalizations, and increased procedural complications. Although guidelines for potassium repletion are available, clinical protocols differ widely across institutions and patient populations, reflecting variability in practice [2].

At the same time, blood transfusion practices in surgical and critically ill patients have become a growing subject of clinical scrutiny. Concerns about the costs, risks, and resource implications of transfusions, particularly adverse immunologic reactions, infections, and increased mortality, have led to stricter transfusion thresholds and individualized decision-making [3]. Numerous factors influencing transfusion requirements have been well documented, such as baseline hemoglobin, hemodynamic instability, intraoperative blood loss, and volume resuscitation strategies. However, the role of electrolyte disturbances, particularly potassium imbalance, and the effects of its correction on transfusion needs have received comparatively little attention in the literature.

Emerging evidence suggests that frequent laboratory monitoring, fluid replacement, and electrolyte correction may contribute to iatrogenic anemia, indirectly influencing transfusion thresholds. Potassium replacement, specifically, can affect cardiovascular dynamics, renal fluid balance, and the intracellular handling of transfused components, all of which may contribute to the need for or avoidance of transfusions [4]. These interactions are especially pertinent in high-risk settings like postoperative recovery and intensive care, where both electrolyte derangements and transfusion decisions are common and clinically significant.

This systematic review aims to synthesize current evidence on the association between potassium replacement practices and blood transfusion outcomes in surgical and critical care patients. The review evaluates both observational and interventional studies that examine potassium supplementation strategies, correction protocols, and their potential influence on transfusion-related endpoints. By systematically exploring these clinical intersections, the review aims to provide meaningful insights that can inform practice guidelines and identify gaps for future research, particularly in optimizing integrated electrolyte and transfusion management strategies.

## Review

Materials and methods

To guide this investigation, a structured Population, Intervention, Comparison, and Outcome (PICO) framework [5] was developed. In brief, the Population included adult patients undergoing surgery or managed in critical care settings; the Intervention consisted of potassium replacement protocols, including intravenous and oral supplementation strategies; the Comparator included items such as standard care, alternative electrolyte protocols, or no supplementation; and the Outcomes focud on blood transfusion rates, transfusion volume, hemoglobin drop, or other transfusion-related metrics.

Search Strategy

A comprehensive literature search was conducted in accordance with the Preferred Reporting Items for Systematic Reviews and Meta-Analyses (PRISMA) guidelines [6]. Multiple electronic databases were searched, including PubMed, Embase, Scopus, and Web of Science, to identify studies published in English that explored potassium replacement practices and their association with blood transfusion outcomes in surgical outcome in cardiac surgery, orthopedic procedures such as total joint arthroplasty, neurosurgical intensive care, and high-volume transfusion settings like trauma surgery, stem cell apheresis and critical care populations. The search strategy combined Medical Subject Headings (MeSH) terms and keywords related to "potassium supplementation," "electrolyte replacement," "blood transfusion," "surgery," and "critical care". Boolean operators (AND, OR) were used to optimize sensitivity and specificity. Reference lists of included articles were also manually screened to identify additional relevant studies. The final selection of articles was guided by relevance to the research objective and conformity to the predefined eligibility criteria.

Eligibility Criteria

Studies were eligible for inclusion if they involved adult human patients (≥18 years) in surgeries like cardiac surgery, orthopedic procedures such as total joint arthroplasty, neurosurgical intensive care, and high-volume transfusion settings like trauma surgery and stem cell apheresis or critical care settings and reported on both potassium replacement practices and transfusion-related outcomes. Eligible studies included prospective or retrospective observational designs, cohort studies, and clinical trials. Articles were required to provide specific data on potassium levels, supplementation protocols, and at least one transfusion-related outcome such as transfusion rate, hemoglobin change, or red blood cell units transfused. Studies were excluded if they focused exclusively on pediatric populations, did not assess transfusion-related outcomes, or did not specify potassium replacement or monitoring strategies. Case reports, editorials, conference abstracts, and non-English publications were also excluded to maintain methodological rigor and ensure data completeness.

Data Extraction

Data extraction was performed independently by two reviewers (SA, MJ) using a standardized template designed to capture relevant study characteristics and outcomes. Extracted variables included study title, first author, publication year, study design, population type, sample size, potassium supplementation protocols (including thresholds, routes, and dosages), comparator groups if applicable, and transfusion-related outcomes. Key findings and relevant contextual notes were also recorded. Any discrepancies in data extraction were resolved through discussion or adjudication by a third reviewer (AAA). This process ensured consistency and minimized potential bias in data interpretation.

Data Analysis and Synthesis

Given the heterogeneity in study designs, patient populations, and outcome measures, a meta-analysis was not performed. Instead, a qualitative narrative synthesis was conducted to integrate findings across the included studies. Thematic analysis was used to identify recurring patterns, such as the prevalence of potassium supplementation, hemoglobin trends, transfusion incidence, and underlying mechanisms linking electrolyte management to blood conservation. Studies were grouped by setting (e.g., surgical vs. ICU) and evaluated for consistency in findings. The strength of evidence was assessed based on study quality, sample size, and clinical relevance, allowing for a contextual understanding of how potassium replacement practices may influence transfusion decisions in high-risk patient populations.

Results

Study Selection Process

**Figure 1:**
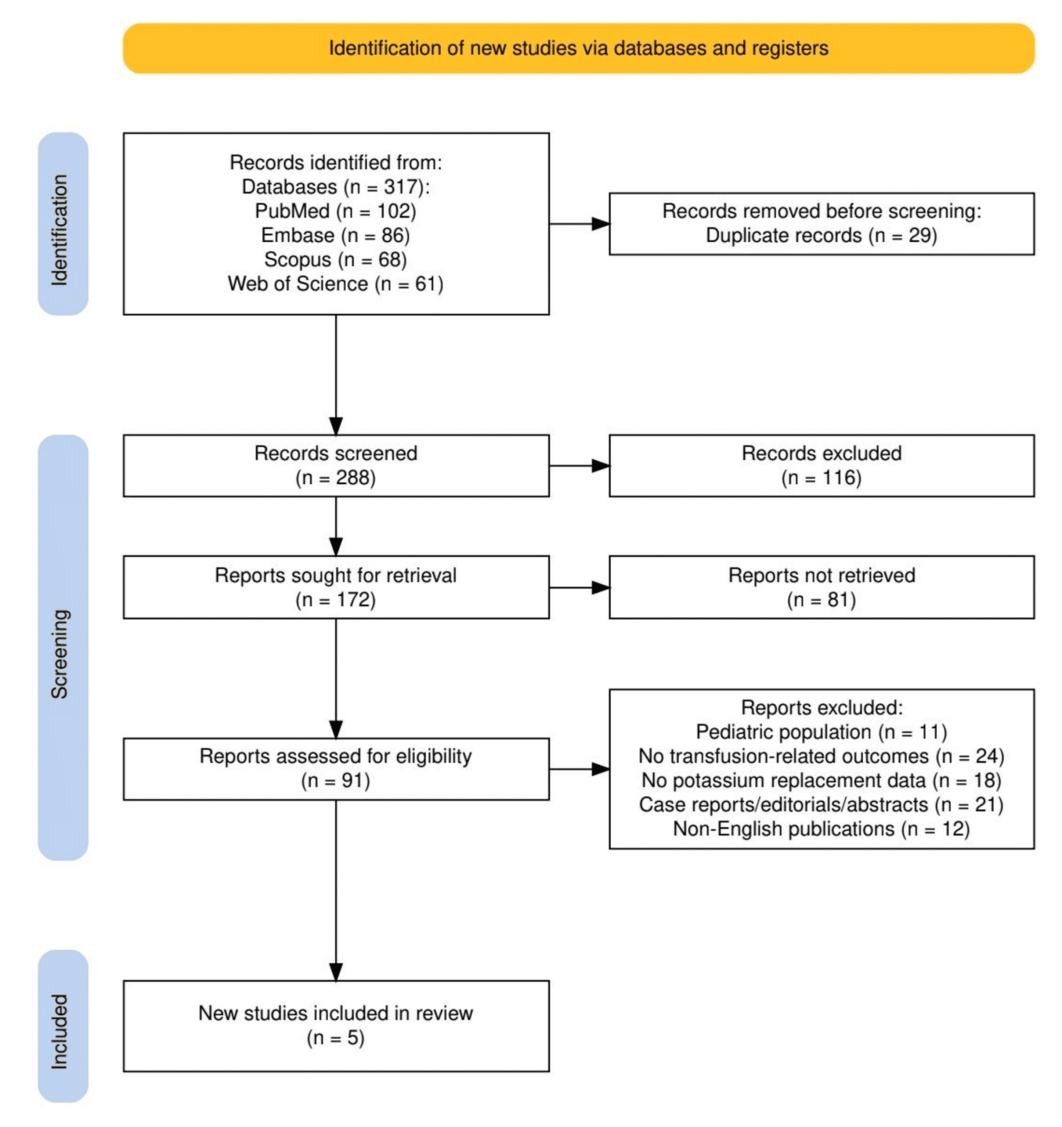
The PRISMA flowchart represents the study selection process. PRISMA: Preferred Reporting Items for Systematic Reviews and Meta-Analyses

The study selection process followed the PRISMA 2020 guidelines and is summarized in Figure 1. A total of 317 records were identified through database searches, including PubMed (n = 102), Embase (n = 86), Scopus (n = 68), and Web of Science (n = 61). After removing 29 duplicate records, 288 unique articles were screened based on titles and abstracts. Of these, 116 were excluded for irrelevance, and 172 full-text reports were sought for retrieval. However, 81 reports could not be retrieved, leaving 91 full-text articles for eligibility assessment. Upon thorough review, 62 articles were excluded due to various reasons, including pediatric populations (n = 11), absence of transfusion-related outcomes (n = 24), lack of potassium replacement data (n = 18), publication type (case reports, editorials, or abstracts; n = 21), and non-English language (n = 12). Ultimately, five studies met all eligibility criteria and were included in the final qualitative synthesis.

Characteristics of the Selected Studies

Table 1 provides an overview of the five studies included in the final analysis, highlighting key methodological and clinical characteristics. The studies encompassed a variety of designs, including prospective and retrospective cohorts, and involved diverse patient populations ranging from individuals undergoing stem cell apheresis to those receiving massive transfusions, critical care, or elective surgical procedures such as spinal and joint arthroplasty. Sample sizes varied substantially, from 60 patients (200 procedures) in specialized apheresis settings to over 1,100 patients in large orthopedic cohorts. Potassium monitoring and replacement strategies differed across studies, with some implementing routine measurements and others providing supplementation based on clinical thresholds or risk stratification. Transfusion outcomes included hemoglobin decline, platelet loss, transfusion incidence, and mortality, offering a comprehensive view of how potassium-related interventions may intersect with blood conservation practices. Notably, the studies demonstrated variability in the presence of formal comparators and protocols, reflecting real-world heterogeneity in clinical management. Despite differing contexts, several studies reported that preoperative or perioperative potassium levels influenced transfusion needs or hemoglobin trends, supporting the review’s central hypothesis.

**Table 1:** Characteristics of the selected studies in the review Hb: hemoglobin; PLT: platelet count; K⁺: serum potassium; Ca²⁺: serum calcium; MIS: minimally invasive surgery; TLIF: Minimally Invasive TLIF: transforaminal lumbar interbody fusion; BMP: basic metabolic panel; ICU: intensive care unit; Pre-op: preoperative; Post-op: postoperative; ↓: decrease

Study (Author, Year)	Design	Population	Sample Size	Potassium Protocol	Comparator	Transfusion Outcome(s)	Key Findings
Schlenke et al., 2000 [7]	Observational (prospective)	Patients undergoing stem cell apheresis	60 patients (200 procedures)	K⁺ measured pre-/post-apheresis; 21% had K⁺ <3.0 mmol/L; >50% needed K⁺ or Ca²⁺ replacement	None	Hb and platelet loss: Hb ↓ 10.7%, PLT ↓ 24.2%	Apheresis causes hypokalemia and Hb drop; >50% required supplementation
Wilson et al., 1992 [8]	Retrospective chart review	Massive transfusion recipients (≥10 units in 24h)	471	Serum K⁺ tracked; 22% hyperkalemia, 18% hypokalemia; pH-corrected K⁺ reduced hyperkalemia to 5%	Survivors vs. non-survivors	Mortality within 48h; K⁺ and Ca²⁺ levels	Higher K⁺ linked to mortality; 94% had low Ca²⁺; aggressive correction recommended
Clark et al., 2011 [9]	Observational cohort	Neuroscience ICU patients (≥5-day stay)	93	Mean 13.1 K⁺ tests/patient; replacement orders recorded; often via full electrolyte panel	Not applicable	Hb drop linked to number of lab draws	Excessive electrolyte monitoring led to phlebotomy-related anemia; conservative testing advised
Ahn et al., 2017 [10]	Retrospective cohort	Patients undergoing 1-level MIS TLIF	332	4.8% received oral K⁺ post-op, mostly due to medication-induced hypokalemia	Low-risk vs. high-risk groups	No transfusions required	Despite Hb ↓, no transfusions; K⁺ replacement rare and symptomless; routine BMP may be unnecessary
Greco et al., 2019 [11]	Retrospective cohort	Primary hip/knee arthroplasty patients	1132	15.5% received K⁺; 72% had pre-op K⁺ <4.0 mmol/L; 28% had comorbidities	Not specified	Transfusion rate: 1.06%	Blood conservation protocol reduced transfusions; pre-op K⁺ <4.0 predicted need for K⁺ or transfusion

Quality Assessment

Quality assessment of the included studies was conducted using the National Institutes of Health (NIH) Quality Assessment Tool for Observational Cohort and Cross-Sectional Studies [12]. As shown in Table 2, all five studies demonstrated acceptable methodological rigor, though the level of quality varied. Two studies were rated as “Fair,” primarily due to limited adjustment for confounding variables and the inherent limitations of retrospective data collection. These studies, despite having clear objectives and reliable outcome measurements, lacked comprehensive strategies to control for potential biases. The remaining three studies were rated as “Good,” reflecting strong design features such as clearly defined populations, appropriate outcome assessment, and adequate sample sizes. These studies incorporated consistent data collection procedures and accounted for clinical stratification, enhancing the validity of their findings. Overall, the body of evidence was considered to be of moderate to high quality, supporting a credible synthesis of findings regarding the association between potassium replacement practices and transfusion-related outcomes.

**Table 2:** Quality assessment of the included studies in the review NIH: National Institutes of Health

Study (Author, Year)	Study Design	Quality Assessment Tool Used	Key Domains Evaluated	Overall Quality Rating
Schlenke et al., 2000 [7]	Observational (prospective)	NIH Tool for Observational Cohort Studies	Clear objective, defined population, reliable outcome measures, but limited confounder control	Fair
Wilson et al., 1992 [8]	Retrospective chart review	NIH Tool for Observational Cohort Studies	Large sample size, defined population, valid measurements, but retrospective and limited in bias adjustment	Fair
Clark et al., 2011 [9]	Observational cohort	NIH Tool for Observational Cohort Studies	Clear inclusion, standard data collection, analysis of hemoglobin outcomes; limited on confounders	Good
Ahn et al., 2017 [10]	Retrospective cohort	NIH Tool for Observational Cohort Studies	Well-defined risk stratification, valid lab data, minimal bias, large cohort	Good
Greco et al., 2019 [11]	Retrospective cohort	NIH Tool for Observational Cohort Studies	Large sample, predefined thresholds, outcome clarity; minor risk from retrospective design	Good

Discussion

This systematic review synthesized findings from five studies evaluating potassium replacement practices and their association with blood transfusion outcomes across surgical and critical care populations. A consistent pattern emerged in which potassium disturbances, particularly hypokalemia, were frequently observed and often addressed through supplementation, although their direct impact on transfusion need varied. 

In stem cell apheresis patients, Schlenke et al. reported that over 50% required potassium or calcium supplementation, with a concurrent mean hemoglobin drop of 10.7%, suggesting a procedural link between electrolyte loss and red cell depletion [7]. Similarly, Wilson et al. found both hyperkalemia and hypokalemia prevalent among patients undergoing massive transfusion, with elevated potassium levels significantly associated with higher mortality, indicating a prognostic relevance in acute settings [8]. In contrast, Clark et al. demonstrated that excessive electrolyte monitoring in neurocritical patients contributed to phlebotomy-induced anemia, indirectly influencing hemoglobin levels and potentially increasing transfusion likelihood [9]. Meanwhile, in surgical settings, Ahn et al. [10] and Greco et al. [11] reported low transfusion rates (0-1.06%) despite mild postoperative declines in potassium and hemoglobin; both studies highlighted the limited clinical necessity for routine lab panels in low-risk patients. Notably, Greco et al. found that preoperative potassium levels below 4.0 mmol/L predicted both the need for supplementation and a higher likelihood of transfusion [11]. While the degree of association varied, the findings collectively suggest that potassium management strategies, particularly when linked to testing frequency or preoperative status, may influence transfusion practices either directly through physiological effects or indirectly via clinical decision-making.

The findings of this review align with a growing body of literature suggesting the need for greater precision in both electrolyte and transfusion management. Current guidelines, such as those from the Association for the Advancement of Blood & Biotherapies (AABB) and National Institute for Health and Care Excellence (NICE), emphasize restrictive transfusion thresholds (e.g., hemoglobin <7-8 g/dL in stable patients) and individualized clinical judgment, yet there remains minimal emphasis on how electrolyte abnormalities may influence these decisions [13]. Similarly, while electrolyte correction protocols, particularly for potassium, are common in ICU and perioperative settings, standardized thresholds for intervention vary across institutions. Our review supports emerging perspectives that routine, non-selective lab testing and correction strategies may not only be clinically unnecessary in low-risk populations but may also inadvertently contribute to hemoglobin decline through excessive phlebotomy. These findings are especially relevant in modern surgical and ICU practices that increasingly emphasize resource stewardship, patient-centered care, and avoidance of unnecessary interventions.

Potassium levels and their correction can influence transfusion-related outcomes through several interlinked physiological pathways [14]. Frequent electrolyte monitoring, particularly when conducted via full laboratory panels, often necessitates repeated blood draws, an underrecognized contributor to iatrogenic anemia, especially in ICU or postoperative patients with limited hematologic reserve. Hypokalemia, if uncorrected, can compromise cardiovascular function and hemodynamic stability, potentially lowering the threshold for transfusion in borderline cases. Conversely, aggressive potassium replacement, particularly in settings of metabolic derangements or renal impairment, may cause fluid shifts or arrhythmic events that complicate overall patient management and impact transfusion decisions. Moreover, the interplay between potassium and other electrolytes (e.g., calcium and magnesium), along with acid-base status, further complicates interpretation, especially in patients undergoing large-volume transfusions or apheresis, where dilutional and citrate-related effects are prominent.

The findings of this review suggest that electrolyte testing and potassium replacement strategies should be more selectively applied, particularly in surgical and ICU settings. Universal protocols that mandate routine electrolyte panels postoperatively or during ICU stays may contribute to unnecessary interventions, including transfusions prompted by test-related anemia or clinically insignificant potassium abnormalities. Individualized testing based on preoperative potassium levels, existing comorbidities, and medication profiles, as observed in some studies, may help identify patients who truly benefit from monitoring and supplementation [15]. Furthermore, the integration of potassium thresholds into clinical decision tools may help avoid redundant testing or treatment, contributing to safer, more cost-effective care. As such, refining potassium replacement protocols could serve as an adjunct strategy to reduce unnecessary transfusions, particularly in resource-sensitive environments.

Strengths and Limitations

This review’s primary strength lies in its exclusive focus on surgical and critical care populations' clinical settings, where potassium disturbances and transfusion decisions frequently intersect. By integrating transfusion outcomes with electrolyte replacement practices, this work highlights a relatively understudied yet clinically relevant interface. The review also benefits from the inclusion of multiple high-quality observational cohorts across diverse clinical contexts. However, several limitations should be acknowledged. Most included studies were observational in nature, limiting causal inference. There was considerable heterogeneity in patient populations, potassium correction protocols, and outcome reporting, which precluded meta-analysis. Standardized transfusion triggers and potassium supplementation algorithms were inconsistently applied or unreported, and data from trauma-specific populations remained notably sparse. These limitations underscore the need for further prospective, standardized research in this area.

Future Direction

Future research should prioritize the design and implementation of prospective studies or randomized controlled trials evaluating the impact of standardized potassium replacement protocols on transfusion outcomes in surgical and critical care patients. Specific attention should be given to trauma cohorts and high-risk ICU populations, where electrolyte imbalances and transfusion decisions are most common and clinically impactful. Additionally, cost-effectiveness analyses examining the potential benefits of limiting routine electrolyte testing could provide valuable insights for institutional guideline development. A harmonized approach to potassium correction based on risk stratification, clinical thresholds, and functional outcomes may offer an opportunity to optimize both resource use and patient safety in high-acuity settings.

## Conclusions

This systematic review highlights a clinically meaningful yet underexplored intersection between potassium replacement practices and blood transfusion outcomes in surgical and critical care settings. While potassium supplementation is commonly employed to correct perioperative and ICU-associated hypokalemia, its role in influencing transfusion needs appears to be both direct through physiological effects on hemodynamic stability, and indirect, via iatrogenic anemia from frequent laboratory testing.

The review highlights the fact that routine, non-individualized electrolyte monitoring and correction may not be necessary in all patient populations and could contribute to avoidable interventions, including blood transfusions. By bringing together evidence from diverse high-acuity environments, this review emphasizes the importance of re-evaluating current potassium management protocols and transfusion practices in an integrated manner. Our findings advocate for more selective, evidence-based approaches that balance clinical vigilance with resource stewardship, an imperative in modern critical care and surgical medicine.
